# Transition From Pulmonary to Gastrointestinal Hemorrhage: A Rare Case of Hematemesis in an Adolescent With Chronic Pulmonary Hypertension and Valvular Regurgitation

**DOI:** 10.1002/ccr3.73480

**Published:** 2026-09-04

**Authors:** Bilal Ashraf, Muhammad Ubaid Hussain, Abdal Zahid, Fatima Sohail, Shan e Ali Shoukat, Modibo Yattassaye, Noreen Ashraf

**Affiliations:** ^1^ Aziz Bhatti Shaheed Hospital Gujrat Pakistan; ^2^ Sahiwal Teaching Hospital Sahiwal Pakistan; ^3^ Jinnah Sindh Medical University Karachi Pakistan; ^4^ Faculty of Medicine and Pharmaceutical Sciences University of Douala Douala Cameroon

**Keywords:** cardiopulmonary hemorrhage, case report, chronic valvular regurgitation, hematemesis, hemoptysis, pediatric pulmonary hypertension, pulmonary hypertension, upper gastrointestinal bleeding

## Abstract

Pulmonary hypertension is a chronic cardiopulmonary disease with increased pulmonary arterial pressure and right‐sided heart overload, and early symptoms often include dyspnea with hemoptysis. Hematemesis, as the presentation of gastrointestinal bleeding, is a remarkably unusual, underappreciated form of the disease, especially in children, along with valvular heart disease. We present a 15‐year‐old boy with preexisting chronic pulmonary hypertension and a history of pulmonary/aortic valvular regurgitation, who reported copious hematemesis after episodes of hemoptysis and epistaxis. At presentation, he was hemodynamically compromised with anemia, hypoxia and features of right‐sided heart strain. Initial work‐up was consistent with an upper gastrointestinal bleed, but further evaluation revealed severe pulmonary hypertension with right ventricular pressure overload and persistent pulmonary and aortic valvular regurgitation on Doppler echocardiography, confirmed radiographically with cardiomegaly and pulmonary vascular congestion. A cardiopulmonary source of bleeding was considered based on the clinical presentation and cardiopulmonary findings, although a gastrointestinal source could not be definitively excluded. The diagnosis was made based on clinical evidence in addition to imaging and echocardiography, consistent with pediatric pulmonary hypertension criteria for the diagnosis. Conservative measures were adopted for the patient, including hemodynamic stabilization, blood transfusion, proton pump inhibitor (PPI) therapy and antifibrinolytics, along with supportive management leading to cessation of hemorrhage and clinical stabilization. This case highlights the need to consider a cardiopulmonary source of bleeding in patients with pulmonary hypertension and hematemesis. Early identification of this unusual presentation is important to prevent misdiagnosis, inappropriate invasive gastrointestinal procedures and timely management with cardiopulmonary resuscitation.

## Key Clinical Message

Hematemesis can represent an atypical manifestation of pulmonary hypertension, particularly in children with coexisting valvular regurgitation. Clinicians should consider cardiopulmonary causes in apparent gastrointestinal bleeding to prevent misdiagnosis, avoid unnecessary invasive procedures, and ensure timely, appropriate hemodynamic and cardiopulmonary management.

## Graphical abstract

Graphical abstract summarizing hematemesis associated with pulmonary hypertension and valvular regurgitation. Negative gastrointestinal evaluation suggested a cardiopulmonary source. Conservative management resolved the hematemesis.

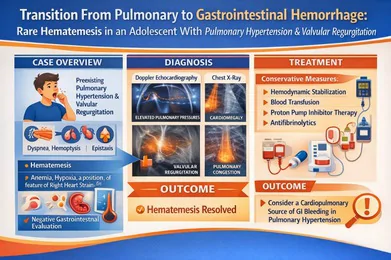

## 1 Introduction

Pulmonary hypertension is a pathophysiological condition defined as abnormally increased blood pressure in pulmonary circulation. Pulmonary hypertension in children is a life‐threatening condition. Complications of PH include syncope, chest pain, and haemoptysis [1, 2, 3]. Hemoptysis is common in pulmonary hypertension due to rupture of hypertrophied bronchial arteries.

Hematemesis has rarely been reported in association with pulmonary hypertension and may create diagnostic uncertainty regarding the underlying source of bleeding [4, 5]. Co‐existing valvular regurgitation may contribute to hemodynamic alterations and venous congestion, potentially increasing susceptibility to hemorrhagic complications. This case report describes a rare presentation of recurrent hematemesis in pediatric patients with pulmonary hypertension and valvular regurgitation, emphasizing the diagnostic and interventional challenges.

Hematemesis reports in pediatric patients with pulmonary hypertension and chronic valvular diseases are unusual. Only a few studies have described atypical bleeding manifestations in pulmonary hypertension, including reports of esophageal varices and other unusual hemorrhagic presentations [6, 7, 8, 9].

## 2 Case Presentation

### 2.1 Patient Information

A 15‐year‐old male, known case of pulmonary hypertension with pulmonary regurgitation and aortic regurgitation, previously presented with hemoptysis, shortness of breath, and epistaxis. The patient had no history of other co‐morbidities such as tuberculosis, asthma, or diabetes. Family history was non‐contributory. The patient belonged to a low socioeconomic background and was a non‐smoker. Detailed characterization of the vomitus and temporal relationship to preceding hemoptysis could not be reliably established.

### 2.2 Chief Complaints

The patient presented to the emergency room with acute hematemesis. He had a prior history of haemoptysis and shortness of breath.

### 2.3 Clinical Findings

On examination, the patient was hemodynamically and vitally unstable. Vital signs were: blood pressure 90/60 mmHg, pulse 110/min, respiratory rate 30/min, and oxygen saturation 90% on room air. General physical examination revealed pallor, anxiety, and dehydration. The patient was conscious and oriented. Cardiovascular examination showed normal S1 and S2 with murmurs of pulmonary regurgitation and aortic regurgitation.

### 2.4 Timeline

A comprehensive timeline summarizing the progression of clinical symptoms, initial presentation with haemoptysis, subsequent development of hematemesis, key clinical evaluations, therapeutic interventions, and subsequent clinical stabilization is provided below (Table 1). This chronological overview facilitates a clearer understanding of the patient's clinical course and highlights the atypical bleeding presentation in the setting of chronic pulmonary hypertension with valvular regurgitation.

**TABLE 1:** Timeline outlining the progression of symptoms, clinical evaluation, management, and outcomes in a patient with pulmonary hypertension and valvular regurgitation.

Time frame	Clinical event
8 November, 2025	Admitted with Shortness of breath, haemoptysis, and epistaxis; Managed in CCU and subsequently discharged
15 November, 2025	Readmitted with recurrent hematemesis. Same day diagnosed as UGIB and managed on IV medications

### 2.5 Diagnostic Assessment

**FIGURE 1:**
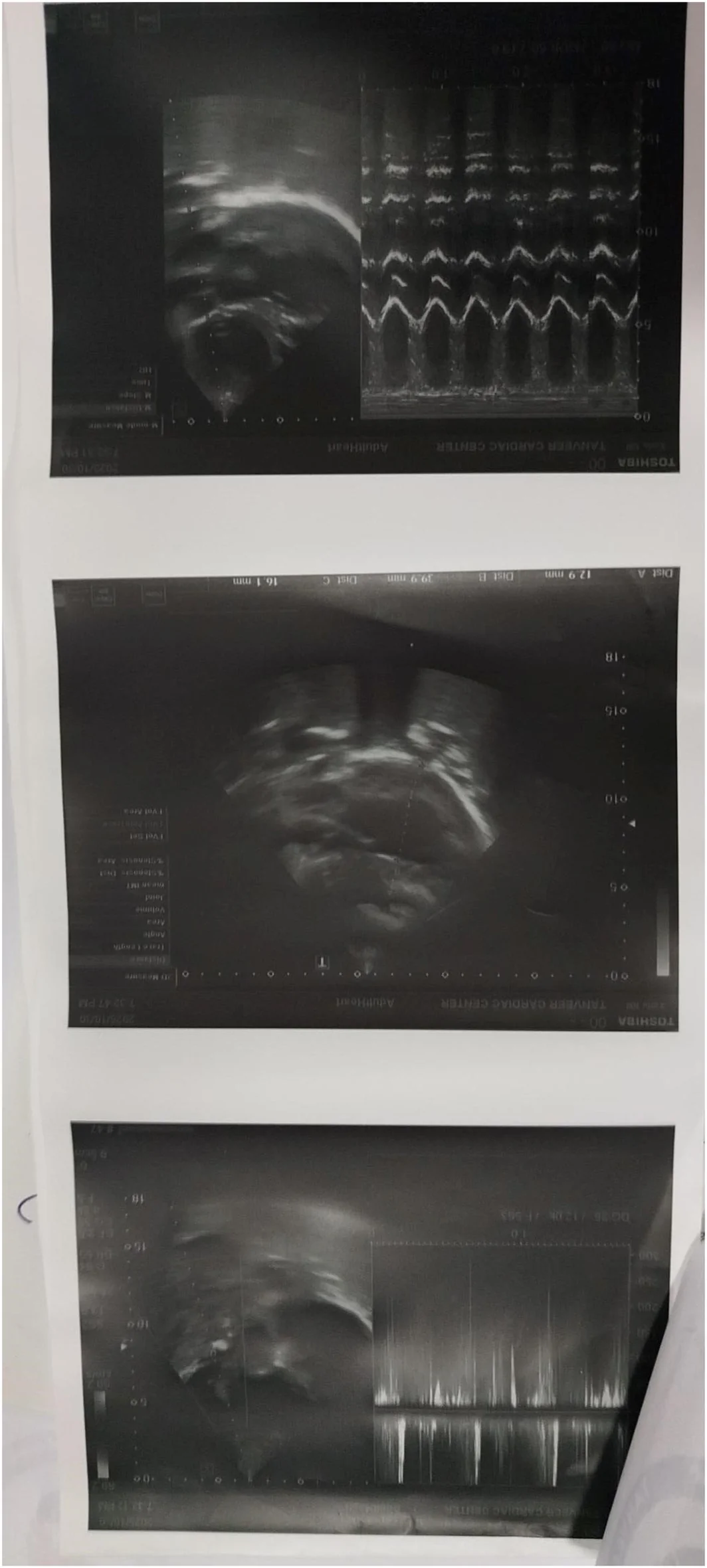
Two‐dimensional (apical four‐chamber view) and Doppler transthoracic echocardiographic images demonstrating right ventricular enlargement, Doppler‐estimated elevated pulmonary artery pressures, and significant pulmonary and aortic valvular regurgitation.

The patient's past medical history was significant for severe pulmonary hypertension with moderate pulmonary and aortic regurgitation, previously documented on transthoracic echocardiography. On current evaluation, repeat echocardiography confirmed severe pulmonary hypertension with right ventricular pressure overload and persistent significant pulmonary and aortic valvular regurgitation (Figure 1).

The echocardiographic images demonstrate right ventricular enlargement, elevated pulmonary pressures on Doppler assessment, and regurgitant flow across the pulmonary and aortic valves, confirming the chronicity and severity of the cardiopulmonary pathology.

**FIGURE 2:**
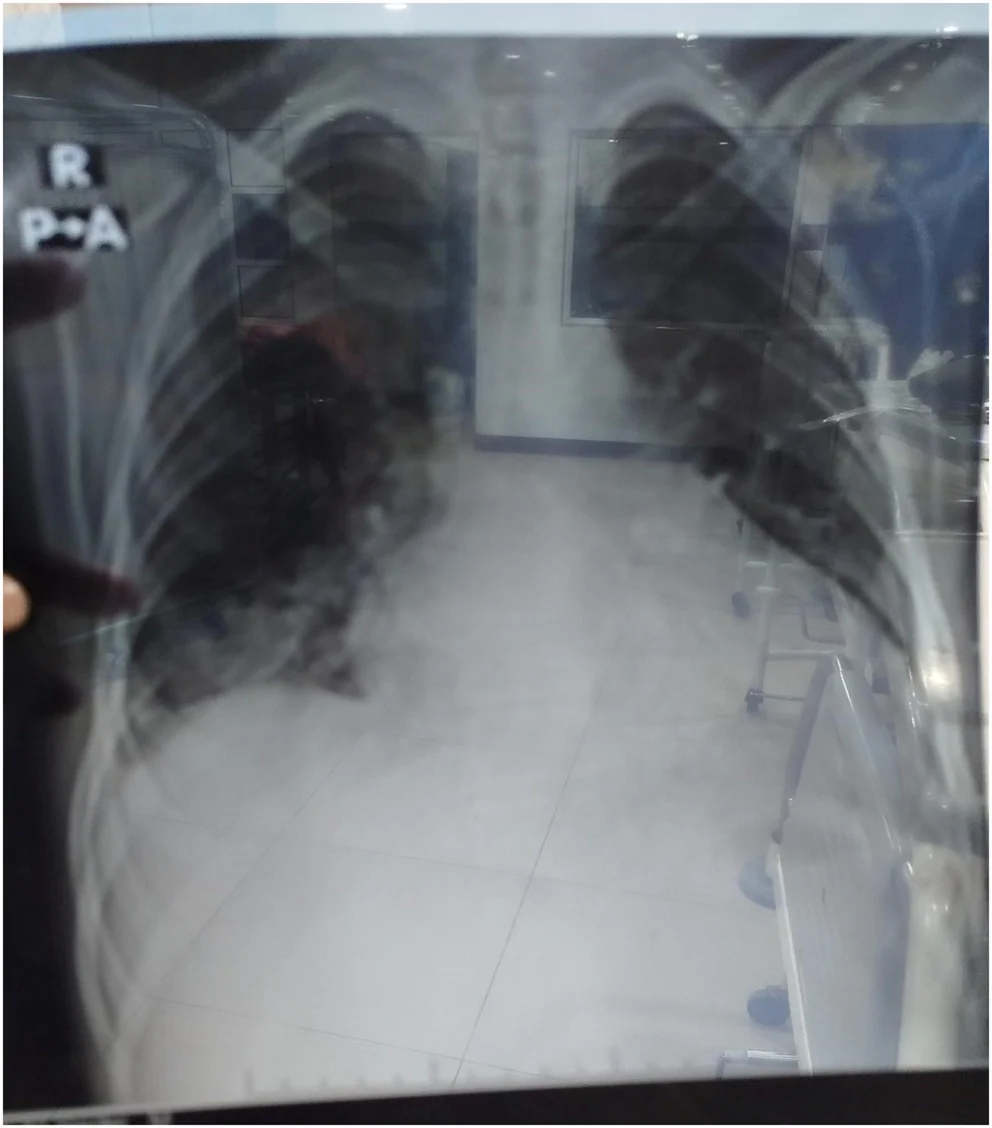
Posteroanterior chest radiograph demonstrating cardiomegaly with prominence of the central pulmonary arteries and increased pulmonary vascular markings, consistent with chronic pulmonary hypertension.

Chest radiography revealed cardiomegaly with prominence of the central pulmonary arteries and increased pulmonary vascular markings, consistent with chronic pulmonary hypertension (Figure 2). The X‐ray findings highlight enlargement of the cardiac silhouette and vascular congestion, corroborating the echocardiographic evidence of severe right‐sided pressure overload.

The patient presented acutely with hematemesis, initially interpreted as upper gastrointestinal bleeding. In the context of underlying severe cardiopulmonary disease, this presentation raised the possibility of a cardiopulmonary source of bleeding in the setting of severe pulmonary hypertension and chronic valvular regurgitation, underscoring the diagnostic challenge in differentiating cardiopulmonary from gastrointestinal sources of bleeding. A cardiopulmonary source of bleeding was considered based on the integration of clinical findings, echocardiographic assessment, radiographic findings, and laboratory evaluation, in accordance with the diagnostic criteria proposed by Mukherjee et al. [10] (Table 2). Which summarizes key clinical and hemodynamic features relevant to pulmonary hypertension and associated cardiopulmonary complications.

**TABLE 2:** Diagnostic criteria for pediatric pulmonary hypertension with valvular regurgitation proposed by Mukherjee et al. [10].

Category	Criteria
Essential Criterion	Elevated mean pulmonary artery pressure (mPAP) > 20 mmHg at rest in children > 3 months of age (hemodynamic threshold)
Major criteria	Pre‐capillary PH hemodynamics: mPAP > 20 mmHg and pulmonary arterial wedge pressure (PAWP) ≤ 15 mmHg and pulmonary vascular resistance index (PVRI) ≥ 3 Wood units × m^2^. Classification of PH sub‐types: (used to refine diagnosis of type): Isolated post‐capillary PH: mPAP > 20 mmHg and PAWP > 15 mmHg and PVRI < 3 WU·m^2^. Combined pre‐ and post‐capillary PH: mPAP > 20 mmHg and PAWP > 15 mmHg and PVRI ≥ 3 WU·m^2^.
Minor (supportive) criteria	Echocardiographic evidence consistent with elevated pulmonary pressures or right heart strain (used when invasive catheter not performed). Clinical symptoms consistent with pulmonary hypertension (e.g., dyspnoea, exercise intolerance, signs of right ventricular overload). Exclusion of other causes of pulmonary hypertension/secondary contributors based on clinical evaluation and baseline testing.
Classification (diagnostic certainty)	Definitive PH: Essential criterion plus hemodynamic major criteria (confirmed by catheterization). Probable PH: Essential + ≥ 1 supportive (minor) evidence when catheterization is unavailable. Possible PH: Elevated mPAP threshold only or strong clinical suspicion pending further testing.

### 2.6 Therapeutic Intervention

The patient was managed as a case of upper gastrointestinal bleeding with intravenous fluid resuscitation, tranexamic acid, hydrocortisone, proton pump inhibitor therapy (esomeprazole 80 mg bolus followed by infusion), and blood transfusion because the patient initially presented with hematemesis. Management followed standard upper gastrointestinal bleeding protocols while further evaluation was undertaken and conservative management was continued.

### 2.7 Follow‐Up and Outcomes

Episodes of hematemesis decreased after intravenous therapy, and the patient became hemodynamically and vitally stable. The primary clinical outcome was cessation of bleeding with restoration of hemodynamic stability. The patient was referred for pediatric cardiology follow‐up. Endoscopy was advised if recurrent episodes occurred. Long‐term management for pulmonary hypertension and valvular regurgitation was planned.

## 3 Discussion

Our patient initially presented with hematemesis in the context of chronic pulmonary hypertension (PH) and valvular regurgitation. While hemoptysis is a common manifestation of PH due to hypertrophied pulmonary and bronchial vessels, hematemesis is exceedingly rare and may be misinterpreted as primary gastrointestinal bleeding, delaying recognition of the underlying cardiopulmonary disorder [1, 2].

Pulmonary hypertension in children is a life‐threatening condition that can arise from multiple causes, including congenital heart defects and chronic systemic involvement [1]. Complications of PH include right ventricular dysfunction, syncope, and pulmonary hemorrhage. In our patient, coexisting pulmonary and aortic regurgitation may have contributed to elevated right‐sided pressures and venous congestion, potentially increasing susceptibility to bleeding in both pulmonary and gastrointestinal circulations [11, 12]. An alternative explanation for the hematemesis is swallowed blood originating from preceding hemoptysis or epistaxis. Given the patient's history of both manifestations, a nasopharyngeal or pulmonary source cannot be excluded. In addition, because upper gastrointestinal endoscopy was not performed, an occult gastrointestinal lesion remains a possible explanation. Therefore, the proposed cardiopulmonary origin of bleeding should be interpreted cautiously.

Several mechanisms may explain the development of hematemesis in this case. Chronic right heart pressure overload can impair venous return from the gastrointestinal tract, leading to mucosal edema and friability. Additional factors, such as stress‐related mucosal injury, coagulopathy from chronic cardiac disease, and swallowed pulmonary blood, may further contribute to gastrointestinal bleeding. The presence of valvular regurgitation amplifies these hemodynamic effects, highlighting the complex interplay between cardiac pathology and atypical hemorrhagic presentations [11, 12, 13, 14].

Diagnosing cardiogenic hematemesis requires careful correlation of clinical, imaging, and laboratory findings. Echocardiography can confirm right ventricular overload and valvular insufficiency, while chest imaging provides evidence of pulmonary vascular congestion [11, 14]. Early recognition allows for targeted management focused on hemodynamic stabilization, correction of anemia, and optimization of cardiac function rather than unnecessary gastrointestinal interventions. Multidisciplinary collaboration among pediatricians, cardiologists, and gastroenterologists is crucial for interpreting such unusual presentations and ensuring timely intervention [12, 13].

This case contributes to the limited literature on atypical hemorrhagic manifestations in pediatric PH. Rare cases have been reported in patients with syndromic or systemic diseases affecting the cardiopulmonary system, where unusual bleeding episodes were observed [11, 12, 13, 14]. Awareness of such presentations is essential for early recognition, prompt management, and prevention of complications in children with complex underlying cardiopulmonary disease.

## 4 Limitations

This report has several limitations. Upper gastrointestinal endoscopy was not performed; therefore, a gastrointestinal source of bleeding could not be definitively excluded. Furthermore, right heart catheterization, the gold standard for diagnosing pulmonary hypertension, was not undertaken. The diagnosis of pulmonary hypertension was based on echocardiographic, radiographic, and clinical findings. Consequently, the proposed pathophysiological mechanism should be interpreted cautiously.

## 5 Conclusion

This case highlights the diagnostic challenge posed by hematemesis in a patient with severe pulmonary hypertension and chronic pulmonary and aortic regurgitation, where a cardiopulmonary source of bleeding was considered. While hemoptysis is the classic manifestation, bleeding that presents as hematemesis can easily be misinterpreted as an upper gastrointestinal source, potentially delaying accurate diagnosis and appropriate cardiopulmonary management. Recognition of such unusual presentations requires a high index of suspicion, careful clinical evaluation, and correlation with imaging and echocardiographic findings.

The coexistence of valvular regurgitation exacerbates venous congestion, potentially increasing susceptibility to hemorrhagic complications in both pulmonary and gastrointestinal circulations. Effective management hinges on prompt hemodynamic stabilization, correction of anemia, and optimization of cardiac function. Multidisciplinary collaboration between pediatricians, cardiologists, and gastroenterologists is crucial to ensure timely intervention, prevent complications, and guide long‐term management.

A limitation of this report is that upper gastrointestinal endoscopy was not performed; therefore, a gastrointestinal source of bleeding could not be definitively excluded. Furthermore, right heart catheterization, the gold standard for diagnosing pulmonary hypertension, was not undertaken.

This case underscores the importance of considering cardiopulmonary causes in atypical gastrointestinal bleeding, particularly in patients with known pulmonary hypertension and chronic valvular disease. Early recognition, vigilant monitoring, and targeted management can prevent morbidity and improve outcomes. Awareness of such rare manifestations is essential for clinicians, as it promotes accurate diagnosis, appropriate therapy, and ultimately, enhanced patient care.

## 6 Patients Perspective

“Before coming to the hospital, I had been struggling with shortness of breath, coughing up blood, and feeling very weak. When I suddenly started vomiting blood, I was scared and confused because I thought something was seriously wrong with my stomach. Learning that it was related to my heart and lungs was shocking. After receiving treatment, I feel much better. I can eat and move around again, and I am grateful to the doctors for identifying the problem quickly and helping me recover.”

## Author Contributions

**Bilal Ashraf:** conceptualization, project administration, supervision, visualization, writing – original draft, writing – review and editing. **Muhammad Ubaid Hussain:** formal analysis, investigation, validation, writing – original draft, writing – review and editing. **Abdal Zahid:** methodology, writing – original draft, writing – review and editing. **Fatima Sohail:** project administration, writing – original draft, writing – review and editing. **Shan e Ali Shoukat:** writing – original draft, writing – review and editing. **Modibo Yattassaye:** project administration, writing – review and editing. **Noreen Ashraf:** project administration, writing – review and editing.

## Funding

The authors have nothing to report.

## Disclosure

*Authorship Statement*: All listed authors meet the International Committee of Medical Journal Editors (ICMJE) criteria for authorship and have approved the final version of the manuscript.

## Ethics Statement

Ethical approval was not required for this case report in accordance with local and institutional guidelines.

## Consent

Written informed consent was obtained from the patient's legal guardian for publication of this case report and accompanying images.

## Conflicts of Interest

The authors declare no conflicts of interest.

## Data Availability

All data supporting the findings of this case report are included within the manuscript.
